# Predictors of Threat from COVID-19: A Cross-Sectional Study in the Spanish Population

**DOI:** 10.3390/jcm10040692

**Published:** 2021-02-10

**Authors:** María del Carmen Pérez-Fuentes, Iván Herrera-Peco, María del Mar Molero Jurado, Nieves Fátima Oropesa, José Jesús Gázquez Linares

**Affiliations:** 1 Department of Psychology, Faculty of Psychology, University of Almería, 04120 Almería, Spain; foropesa@ual.es; 2 Nursing Department, Faculty of Health Sciences, Alfonso X El Sabio University, 28691 Madrid, Spain; 3 Alfonso X El Sabio Foundation, 28691 Madrid, Spain; 4 Department of Psychology, Universidad Autónoma de Chile, Providencia 7500000, Chile

**Keywords:** coronavirus, COVID-19, public mental health, quarantine

## Abstract

One of the first measures for fighting the worldwide spread of the COVID-19 pandemic is social isolation or quarantine. The perceived threat from COVID-19 in this situation, maintained over time, generates uncertainty and fears, etc., which could lead to mental disorders in the population. This study evaluated the perceived threat from COVID-19 in the Spanish population. The study design was cross-sectional and observational. The sample of 1014 participants recruited in Spain had a mean age of 40.87 (*SD* = 12.42). The gender distribution was 67.2% (n = 681) women and 32.8% (n = 333) men. Data were collected with an online survey. The instrument used was the Perception of Threat from COVID-19 Questionnaire, validated for the Spanish population. Our data showed a clear correlation between perceived threat with female gender, having children in one’s care and level of education. However, no association was observed with age or marital status. Finally, we concluded that there is a greater perception of threat from COVID-19 by women with a lower education who have children in their care, and that they are also more sensitive to minor mental disorders, such as anxiety or stress, appearing.

## 1. Introduction

When pneumonia was detected in December 2019 in Wuhan, Hubei Province, China, its origin was unknown. However, by the beginning of January 2020, it was identified as the novel SARS-CoV-2 virus which causes the disease known as COVID-19 [1,2,3]. Its course goes through a series of systemic physical symptoms, such as fever, cough, fatigue, headache and diarrhea, and also respiratory affections that could include rhinorrhea, pneumonia or acute respiratory distress syndrome [3,4,5]. 

COVID-19 characteristics facilitated its rapid expansion, leading the World Health Organization to define it as a global pandemic on 31 January 2020 [6]. Its incubation period is about 5.2 days [7], with symptoms appearing in an average of 14 days [8]. In addition, a high percentage of virus carriers are asymptomatic, but they are nevertheless infective and can infect others if not detected in time [9], which, along with the enormous stream of transportation, could amplify its spread [10,11] and the danger it represents to public health [12].

Due to the spread of the virus and the disease it causes, as well as the inexistence at the present time of effective treatments or vaccination against SARS-CoV-2 [13,14], a number of measures have been taken to reduce its spread and protect the population. These may be grouped in two main measures: limiting movement of the population and home confinement [15,16]. These measures, in addition to the pandemic itself, can have effects, not only on particular individuals but also on the physical and mental health of the entire population [17,18,19,20,21,22], and especially frontline professionals, such as healthcare workers [23,24]. With respect to the COVID-19 disease itself, as described in other infectious disease epidemics [25,26,27,28], some people have negative emotions causing behavior and attitudes that cause them to avoid contact with disease [29]. This defensive reaction to perceived threat from the disease can cause severe psychological maladjustments such as stress, depression and anxiety [17,20,26,30].

The confinement due to the COVID-19 pandemic has been described by some authors as a possible cause of collective hysteria [31,32,33], a situation which, if it worsens and measures are hardened to mass quarantine, could generate anxiety [18]. It has been observed that people subjected to isolation may experience feelings of loneliness and anger in addition to problems in relating to others person-to-person and even in group social relations when isolation ends [33,34].

The unpredictability of information received from authorities on control of the disease or severity of risk of contagion, and disinformation from both traditional communication media [18,35] and on social networks such as Twitter, YouTube, Instagram or Facebook, among others [36,37,38] combine with the situation above, generating stress, fear, guilt, displeasure [18,34,35,38] and so forth. Although they may not be considered mental illnesses in themselves, they can lead to situations compromising mental health [35]. Therefore, one’s perception of the disease depends on the interpretation of experiences, how that interpretation is transferred to active behavior, the response to social reactions and the personal meaning attributed to the experience [39]. In the situation of imminent alarm in which society around the world now finds itself with COVID-19 and its effect on health [16,17,24], the perception adults have of the disease as government measures change their habits, becomes very important. Perhaps one of the most significant changes is in the care of children or other dependents [40], as women, who traditionally care for the most vulnerable members of the family [41,42,43], could find their situation worsened under conditions such as those generated by the current pandemic. 

The significant role of healthcare professionals as guarantors of both physical and mental health of the population [44], even in situations of public health conflict [45] should also be mentioned. At the present time, there is not much information on the psychological impact and mental health of the general population [6]. A large number of scientific publications have focused on analysis and identification of epidemiological and clinical characteristics of infected patients, genome identification and morphology of the virus and situations related to the logistics and political and healthcare policy decision-making [5,32,46]. The the psychological state of the Spanish population has not yet been defined, although there are such studies on specific groups in the Spanish population, like nurses [47] or university students [48]. 

The objective of this study was to explore the threat perceived by Spanish society from the lockdown imposed because of the COVID-19 epidemic. We think that uncertainty and lack of information about COVID-19 could affect cognitive and emotional health [7]. An evaluation of perceived threat by COVID-19 [39] that would provide information on which groups are the most sensitive to the pernicious effects on mental health of both COVID-19 and the measures taken to slow down its contagion would be useful for healthcare authorities as well as primary care professionals in attending patients. 

## 2. Method

### 2.1. Participants

The study sample was made up of a total of 1043 Spanish adults residing in 19 autonomous regions of which Andalusia was most represented with 37.9% of the participants, followed by Madrid with 27.5%. Of these 29 were eliminated because of random or incongruent answers on control questions included in the questionnaire, leaving 1014 participants in the study. 

Ethical research standards were complied with, providing information on the project and requesting consent to participate. The study was approved by the University of Almería Bioethics Committee. 

### 2.2. Design and Data Collection 

A cross-sectional observational study planned was carried out as an online survey due to the state of emergency decreed in Spain last 14 March and the restriction of movement, making a person-to-person format impossible.

The sample was acquired by snowball sampling by spreading the link to the questionnaire on social networking sites. Data was acquired from 18 March through 23 March 2020.

### 2.3. Instruments and Variables

This study used the Perception of Threat from COVID-19 Questionnaire validated for an adult Spanish population [39]. The questionnaire consists of five items focused on the perception of threat from COVID-19 (Table 1), where the participants rate their agreement with the statements on a Likert-type scale of 0 to 10. The test offers an overall score on the representation of the disease, where the highest scores indicate greater perception of COVID-19 as a threat. This questionnaire showed acceptable internal consistency (α = 0.66).

In addition, an ad hoc questionnaire on the following socio descriptive variables was included: Gender (man or woman), age, marital status (married, single, widowed or divorced), education (no education, primary school, high school and higher education), autonomous region, “Do you have any minor children?” (yes, no), “Is anyone close to you COVID-19 positive?” (yes, no).

### 2.4. Data Analysis 

First, relative and absolute frequencies were calculated in a descriptive analysis of the sociodemographic variables. 

Then, relationships between the quantitative variables were explored by correlation analysis, and categorical variables by Student’s *t*-test and ANOVA. In the hypothesis comparisons, 0.05 was considered statistical significance and the confidence intervals were calculated at 95%.

After that, a binary logistic regression was performed using the enter method. The dependent variable for this was perceived threat, previously dichotomized into medium-low/medium high. The predictor variables, based on the results of preliminary analyses, were sex, having minor children in one’s care and education. 

SPSS version 23.0 for Windows was used for data processing and analysis.

## 3. Results

### 3.1. Descriptive Analysis

The mean age was 40.87 (SD = 12.42) in a range of 18 to 76. The gender distribution was 67.2% (n = 681) women and 32.8% (n = 333) men, with a mean age of 39.88 (SD = 12.35) and 42.92 (SD = 12.33), respectively. Over 90% of the sample was single (30.9%) or married (60.1%). Over 90% had a secondary or higher education (16% and 78.7%, respectively). When asked if they had minor children, 35.9% (364) answered affirmatively (see Table 2). And finally, only 16.4% (n = 166) had someone COVID-19 positive close to them.

Potential explanatory variables were selected by descriptive analysis of their relationships with perceived threat. The quantitative variables were examined with bivariate correlations, in which no correlation with perceived threat was found for age: *r* = 0.05, *p* = 0.092, 95% *CI* (−0.009; 0.114). Education, coded on a scale in ascending order from 0 = “no education” to 3 = “higher education”, correlated negatively to perceived threat: *r* = −0.08, *p* < 0.01; 95% *CI* (−0.149; −0.027). No statistically significant between-group differences were observed by marital status (*F* = 2.03; *p* = 0.108) in the analysis of perceived threat.

**Figure 1:**
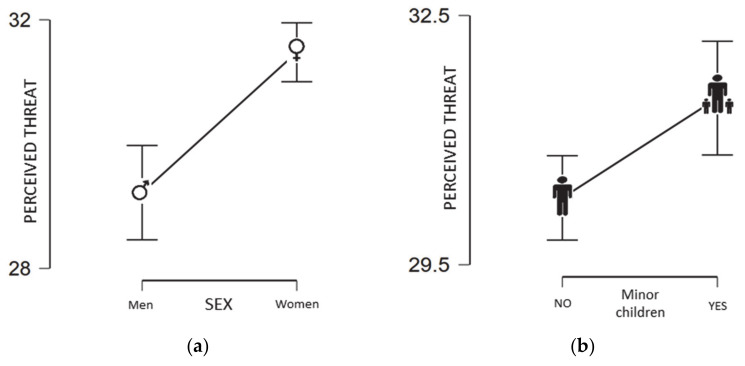
(**a**) Perceived threat by gender. (**b**) Perceived threat by whether there were minor children in their care.

However, differences were detected (t1012 = −5.15; *p* < 0,001; d = 0.34) by gender (Figure 1a), in which women perceived higher threat (M = 31.47; SD = 6.29) than men (M = 29.21; SD = 7.03). Furthermore, those with minor children in their care (M = 31.50; SD = 6.63) differed significantly (t1012 = −2.77; *p* < 0,01; d = 0.18) from those who did not (M = 30.30; SD = 6.59), where the first scored higher in perceived threat from COVID-19 (Figure 1b).

### 3.2. Logistic Regression Model

Based on the above descriptive analyses, the independent variables entered in the logistic regression model for predicting perceived threat were gender, having minor children and education. In this case, the total score for the variable on the questionnaire was previously dichotomized by visual grouping and percentiles based on the cases explored. 

The cutoff point was set at 31.5, forming two intervals or groups, one medium-low threat with scores equal to or lower, and medium-high threat, with higher scores. Later recording of the variable (once the cutoff points had been found by visual grouping) was done manually.

Table 3 shows the results of the logistic regression analysis: regression coefficients, standard error of the estimate, Wald statistic, degrees of freedom and associated probability, partial correlation coefficient and cross-product.

The odds ratio found for each variable indicates that risk of perceiving strong threat is higher among women with minor children in their care and with low education.

Overall fit of the model (χ^2^ = 32.57; df = 3; *p* < 0.001), was confirmed by Hosmer–Lemeshow test (χ^2^ = 1.54; df = 5; *p* = 0.908). In addition, the Nagelkerke R2 indicated that 4.2% of the variability in the response variable would be explained by the logistic regression model. 

## 4. Discussion

The novel results of this study found possible psychological problems related to perceived threat from the infectious disease COVID-19. 

In the first place, analysis of the threat perceived by the population showed that neither participant age nor marital status influenced perception of threat. However, gender did influence that perception. It was observed that women were particularly more prone to perceive the COVID-19 disease as a threat. This might be attributed to a woman’s traditional role in society as planner and caregiver of the family unit [41,42,43], related to a feeling of moral and affective obligation [40]. Although men have become more involved with childcare since the economic crisis of 2008, or when they are unemployed, it seems that this trend is not maintained when they are employed, devoting less time to caring for children than mothers [40]. This is not the case of women, who care for the family regardless of whether they are otherwise employed. 

Another factor found to be positively related to perception of threat was having children in one’s care, which could be associated with fear that the children would be infected by the disease or even lost [23,28,49].

A lower level of education was associated with perceived threat from COVID-19, perhaps related to access to sources of information and to understanding based on previous knowledge [47,48,50]. Thus, a higher level of education would be associated with a greater critical capacity of information consumed and processed and the tendency to seek other sources of information to corroborate or refute information acquired [51]. 

It is worth mentioning that information sources, whether communication media or social networking sites, may generate uncertainty [18,35,36,38] because of the way the news is explained, providing incoherent data which could generate anxiety or fear in an epidemic, or by way of “false experts” who generate biased and erroneous interpretations of data, causing confusion and unease. 

A clear example that social networks can generate a high percentage of untrustworthy information if one does not know how to filter it is YouTube, where during the Zika pandemic, it was found that 25% of the videos published on that subject contained unreliable and biased information [38]. This was also true during the Ebola pandemic, where 63.5% of the videos analyzed contained unreliable information [37], and also at other social networking sites [36]. This situation of uncertainty due to access to unclear and even biased information can generate a high level of uncertainty associated in turn with anxiety and depressive symptoms [20].

In addition, it was found that women without an education and with minor children in their care had a stronger feeling of threat from COVID-19. This could explain the association in the sample studied, as they did not have enough knowledge to enable them to filter information received from the communication media or social networks, thus generating anxiety and stress, a normal response of fear and protection for loved ones [19,49] in the traditional caregiver role of women [40,42,43].

Even though the COVID-19 pandemic is considered a public health emergency [12] understood as a binomial made up of physical and mental health [21], it should be highlighted that there are no studies on the analysis of threat perceived by the population and the possible importance of this perception on development of alterations in mental health during crisis situations, such as the COVID-19 pandemic. In this regard, the increase in minor mental disorders in the Spanish population during the economic crisis of 2008 should be emphasized [44]. This situation and experiences in countries where the fight against the disease has been longer, such as China, makes intervention for possible psychological affectation necessary in the population as a public health response [18,22,25]. 

In spite of the contributions made in this study, it is important to emphasize its limitations. The study sample, due to the nonprobability sampling used, was not representative. In this respect, it should be mentioned that a high percentage of participants were women, and that most of the participants had a higher education, which also affects the representativeness of the results. Moreover, there may have been social desirability biases associated with the self-reports used for data collection. Lastly, (although it might not be considered a real limitation, it should be noted that) due to the sudden occurrence of the pandemic, we were unable to assess the mental health burden in a Spanish population beforehand. Therefore, future research should improve the sampling technique to avoid possible biases. 

Finally, while our original research goal was to analyze the perception of the threat associated with the COVID-19 pandemic and control strategies for reducing the spread of the virus, we realize that previous studies have also been done in countries like Italy [52], Greece [53] or Canada [54] that suggest a relationship between the COVID-19 pandemic and control strategies, with the appearance of anxiety and depression disorders in these populations. Therefore as a future line of research, we will delve more deeply into the relationship of anxiety and depression to the Spanish population’s mental health during the COVID-19 pandemic.

## 5. Conclusions

In conclusion, the results of this study show that in a situation such as the one we are now experiencing, there is a feeling of threat from COVID-19, which is worsened by isolation during lockdown. Some groups in the sample had a greater perception of threat, especially women with lower education who have children in their care, and they were more sensitive to minor mental disorders appearing, such as anxiety or stress. 

We believe this situation may be similar to past economic crises which have caused a significant increase in burnout [54] and mental disorders in Spain. Therefore, healthcare authorities should evaluate the implementation of policies directed at providing the material and human resources for healthcare professional teams in community care, so these professionals can detect and act quickly against any minor mental health disorder derived from the stress and fear from perceived threat of COVID-19 and daily abnormal situations through community activities and even educational intervention. 

## Figures and Tables

**Table 1:** Items measured by the Perception of Threat from COVID-19 instrument (by author).

Items	Range	M	SD
How much is coronavirus infection affecting your life?	1–10	7.62	2.13
How long do you think the coronavirus infection alert will last?	1–10	6.84	1.60
To what extent do you feel symptoms due to infection by coronavirus?	1–10	2.03	1.85
How much are you worried about infection by coronavirus?	1–10	7.65	2.15
How much are you affected emotionally by infection by coronavirus? (That is, do you feel furious, afraid, angry, depressed?)	1–10	6.58	2.41

**Table 2:** Sociodemographic characteristics of the participants.

Variables	%	n
Sex		
Men	32.8%	333
Women	67.2%	681
Marital status		
Single	30.9%	313
Married	60.1%	609
Divorced	8.1%	82
Widowed	1%	10
Education		
No education	0.3%	3
Primary school	5%	51
High school	16%	162
Higher education	78.7%	798
Do you have any minor children?		
No	64.1%	650
Yes	35.9%	364
Is anyone close to you COVID-19 positive?		
No	82.6%	848
Yes	16.4%	166

**Table 3:** Results derived from the logistic regression for probability of perceived threat.

Variables	β	St. Error	Wald	df	Sig.	Exp(β)	CI 95%
Sex (a)	0.633	0.137	21.224	1	0.000	1.884	1.439–2.467
Minor children (b)	0.355	0.134	6.980	1	0.008	1.426	1.096–1.857
Education	−0.261	0.116	5.031	1	0.025	0.770	0.613–0.968
Constant	0.159	0.341	0.217	1	0.641	1.172	

Note. (a) Women; (b) With minors in their care.

## Data Availability

The data presented in this study are available on request from the corresponding author.
